# Diet Switching from Leaves to Flowers – is this Beneficial for the Mustard Leaf Beetle?

**DOI:** 10.1007/s10886-025-01668-1

**Published:** 2025-11-27

**Authors:** Kristina Runte, Dominik Ziaja, Caroline Müller

**Affiliations:** 1 Bielefeld University, Universitätsstr. 25, Bielefeld, 33615 Germany; 2 Joint Institute for Individualisation in a Changing Environment (JICE), University of Münster and Bielefeld University, Bielefeld, Germany

**Keywords:** Choice assays, Diet mixing, *Nasturtium officinale*, Niche choice, Performance, Herbivore preference

## Abstract

Many herbivorous species are considered specialists that feed not only on specific plant taxa, but also on certain organs. Numerous species feed on leaves (folivory), while supplementary feeding on flowers (florivory) or switching in diets is less commonly considered. We used the mustard leaf beetle, *Phaedon cochleariae*, known to feed on leaves of Brassicaceae, such as *Nasturtium officinale*, to test whether it also accepts flowers, whether different stages show different preferences for certain plant organs, how the glucosinolate contents of the plant parts differ and how individuals develop on either single or mixed diet. In preference tests, larvae and adult males did not differentiate between young and old leaves or leaves versus flowers, but rarely accepted fruits. Adult females preferred young over old leaves and leaves over flowers, while they did also accept fruits. Total glucosinolate concentrations were higher in young leaves and flowers than in old leaves and fruits. In development assays with four different groups of diets, larvae that were exclusively fed on leaves or switched to flowers over the larval development developed faster than those only fed on flowers. However, exclusive feeding on leaves led overall to the highest fertility, while individuals fed exclusively on flowers showed by trend the lowest survival. Since *P. cochleariae* can apparently also feed and develop on flowers, a switch to this organ may be beneficial if leaves turn senescent or to exploit a competition- or enemy-free niche. Partial florivory amongst species considered to be folivores may be more widespread than assumed.

## Introduction

Many herbivorous insects are feeding specialists, using only one or a few species within a plant family as hosts. Moreover, herbivorous insects are usually considered to be adapted to feed on a certain plant organ, such as the leaves (folivores) or the flowers (florivores) (Schoonhoven et al. 2005; McCall and Irwin 2006). However, a few studies demonstrated that species long considered as classical folivores also feed on flowers or switch during their ontogeny from folivory to florivory, such as, for example, caterpillars of *Pieris brassicae* (Lepidoptera: Pieridae) (Smallegange et al. 2007) or larvae of *Athalia rosae* (Hymenoptera: Tenthredinidae) (Bandeili and Müller 2010). In these two species, larvae begin moving from the leaves to the flowers of their host plants, *Brassica nigra* and *Sinapis alba*, respectively, from the late-second (*Pi. brassicae*) or third instar (*A. rosae*) onwards. Moreover, individuals of *Pi. brassicae* and *A. rosae* feeding exclusively on flowers develop faster and reach a higher body mass than individuals kept exclusively on leaves (Bandeili and Müller 2010; Smallegange et al. 2007). Larvae of the viburnum leaf beetle *Pyrrhalta viburni* (Coleoptera: Chrysomelidae) feed preferably on young leaves, but when those are absent, also feed on inflorescences (Desurmont and Blanchet 2023). However, an exclusive diet on flowers has negative consequences for their survival probability and leads to a prolonged development time and lower body mass compared to individuals raised on young leaves. Opportunistic florivory may thus be an alternative when leaves are not available and has advantages due to the higher nutritional quality and better digestibility of flowers compared to leaves, at least in some species (Burgess 1991; Matter et al. 1999; Thompson 1983). However, for numerous herbivorous species considered as folivores, little is known about potential florivory and whether this may benefit individuals in the short-term and populations in the long-term.

Diet mixing may complement and balance the nutrient intake, while also diluting high concentrations of potentially toxic specialized metabolites (Hägele and Rowell-Rahier 1999; Raubenheimer and Simpson 1999; Singer et al. 2002). Learning and chemoreceptor modulation can further modulate diet mixing of individuals (Bernays and Bright 1993). Impacts of diet mixing have mostly been studied in generalists and with different plant species as food source (Desurmont and Weston 2014; Hägele and Rowell-Rahier 1999; Singer et al. 2002), but also applies in specialists that use variation within the host plant species by mixing plant genotypes or different plant parts (Mody et al. 2007; Tremmel and Müller 2013). Individuals may also integrate and overcome qualitative differences between different dietary sources by utilizing the resources obtained from a previous diet for growth when faced with a poorer quality diet (Tremmel and Müller 2013). Through diet mixing, individuals of some species speed up their development (Moreau et al. 2003) or reach a higher fecundity (Merwin and Parrella 2014; Mody et al. 2007) compared to conspecifics that feed on just one diet. However, diet mixing is not always beneficial, and the outcome highly depends on the nutritional quality of the diet or mechanical and chemical defense of the different plants (Friedrichs et al. 2022a). In such experiments, individuals are usually given either a constant single diet or a constant mixture of diets that are offered together, but they rarely receive the food in sequence. However, findings such as those described above for *Pi. brassicae* and *A. rosae* demonstrate that a switch to another diet, which can be just another organ of the same plant species, may also depend on the herbivores’ ontogeny (Bandeili and Müller 2010; Smallegange et al. 2007), leading to sequential feeding of different diets. After their first feeding experience, neonate larvae may also become hooked on certain metabolites that form a recognition template, as shown for different specialist species (Renwick and Lopez 1999; del Campo et al. 2001). Thus, they may not accept food lacking these compounds, preventing them from diet mixing later in their development.

Within host plant species, primary and specialized (also called secondary) metabolites, playing a role in insect nutrition or acting as potential deterrents or toxins, respectively, are differently distributed (Stamp 2003; Schoonhoven et al. 2005; Bandeili and Müller 2010). According to the optimal defense theory (Stamp 2003), tissues and organs that are of particular high value for a plant individual, such as its reproductive organs, should be better defended than less valuable plant parts. In line with this theory, higher defense levels were found in flowers than in leaves of several plant species, but this is not a general pattern (McCall and Fordyce 2010). Traditionally, leaves have also been much more studied for their defenses than flowers (Oguro and Sakai 2014). In Brassicaceae, glucosinolates are the characteristic specialized metabolites that are converted into toxic metabolites upon reaction with myrosinase enzymes stored in separate compartments within the tissues (Wittstock et al. 2016). The intra-individual variation in glucosinolate concentrations differs between plant species. For example, in *Brassica nigra* substantially higher glucosinolate concentrations were found in flowers compared to leaves (Chrétien et al. 2022). In contrast, comparable glucosinolate concentrations between flowers and young leaves were present in other Brassicaceae species, such as *Sinapis alba* and *Lepidium draba* (Bandeili and Müller 2010; Saadellaoui et al. 2025), but old leaves of *S. alba* had much lower glucosinolate concentrations (Bandeili and Müller 2010).

The mustard leaf beetle, *Phaedon cochleariae* (Coleoptera: Chrysomelidae), is a feeding specialist on different Brassicaceae plants, developing particularly well on watercress (*Nasturtium officinale*) (Reifenrath and Müller 2009). In the field, both larvae and adults are usually found on leaves, but occasionally, they are found on the flowers, especially when mating; sometimes they also occur in high densities on the plants (A. Barber, personal communication). Adults were found to prefer young over old leaves of another Brassicaceae, Chinese cabbage (*Brassica rapa* ssp. *pekinensis*), when having had prior experience with young leaves (Tremmel and Müller 2013). Adult beetles naïve to *N. officinale* also preferred green over yellow color in general, and more specifically, young green over old yellow leaves of this plant species. Furthermore, females laid more eggs on young leaves (Kühnle and Müller 2011). When reared on young leaves of cabbage, larvae reached a higher body mass and developed faster than when reared on old leaves (Müller and Müller 2017). Larvae reared on a mixed diet of young and old leaves offered in alternating order (switched every two days) developed as fast as those reared on young leaves only and had a comparable efficiency of conversion of food (i.e. body mass gain in relation to consumed food) (Tremmel and Müller 2013). Young cabbage leaves also had higher glucosinolate concentrations and carbon and nitrogen contents compared to old leaves, while the carbon-to-nitrogen ratio did not differ (Müller and Müller 2017; Tremmel and Müller 2013). To our knowledge, the acceptance of – or performance on – flowers or fruits by *P. cochleariae* has not been tested. However, beetles of *Phaedon brassicae* are known to feed on flowers and flower buds of *Arabidopsis halleri*, potentially selecting for early flowering time in this plant species (Kawagoe and Kudoh 2010). With regard to plant defenses of Brassicaceae, both larvae and adults of *P. cochleariae* are able to cope well with the hydrolysis products of various glucosinolates by conjugating them with amino acids (Barber et al. 2024; Friedrichs et al. 2022b). For example, feeding of a diet containing 2-phenylethyl glucosinolate, the major glucosinolate in *N. officinale*, results in the formation of *N*-(phenylacetyl) aspartic acid, which is excreted with the feces (Friedrichs et al. 2020).

The aim of this study was to investigate the feeding preferences of *P. cochleariae* for different plant parts of the host plant *N. officinale* and to test whether larvae and adults accept flowers or fruits for feeding, and, in the case of females, for oviposition. The organs were analyzed for their glucosinolate concentration and composition, as these may affect the preferences. Furthermore, we aimed to study the developmental and reproductive performance in dependence of the diet, i.e. when individuals were fed exclusively with leaves or with flowers, or with a sequence of initially leaves for the first four or eight days after larval hatching and then flowers (i.e., four treatment groups). We hypothesized that larvae and adults will also accept flowers and that a diet mix will be beneficial for the development and reproduction. We furthermore hypothesized that glucosinolate concentrations are highest in flowers of *N. officinale* as the most valuable tissue.

## Materials and Methods

### Plants and Insects

Batches of plants of *N. officinale* (seeds from Volmary GmbH, Münster, Germany) were weekly grown in pots (12 cm diameter, 9 cm height, ca. 50 seeds per pot) in a greenhouse at a 16:8 L: D cycle, ca. 20 °C, and 60% r.h. on soil (HAWITA-Fruhstorfer type T, with 2 cm top layerof type P soil). As *N. officinale* is a helophyte, the pots were standing in trays filled at least 2 cm with water. The plants were six to eight weeks old when used for the experiments. At that age, the shoots have leaves of all developmental stages as well as flowers and first fruits and are between 15 and 25 cm tall. In nature, the plants flower from end of May to July (Düll and Kutzelnigg 2016).

Individuals of *P. cochleariae* were kept in a laboratory culture, which was yearly mixed with beetles collected from the field. In that way, the rearing has been already maintained for more than ten years. Insects were kept in boxes (20 × 20 × 6.5 cm) with approx. 100–200 individuals per box, placed in a climate cabinet with 16:8 L: D cycle, 20 °C and 65% r.h. and provided with stems of *N. officinale* carrying leaves, flowers and fruits, which were refreshed every other day. The boxes were cleaned once a week.

### Preference Experiment

To test whether larvae and adult males and females show different preferences and accept also other plant parts apart from leaves, individuals were placed in Petri dishes (5.5 cm diameter) lined with moistened filter paper and offered two different plant parts of *N. officinale*. Larvae were tested eight days after hatching and a starvation period of 1.5 h, while adults were tested at an age of 39–47 days and a starvation period of 4 h. Time periods were based on previous experience; a certain degree of hunger was induced without the individuals suffering too much from nutritional deficiency. Individuals were offered a choice between leaf discs (10 mm diameter, cut with a cork borer) of a young leaf (from the upper part of a stem) and an old leaf (still green, from the lower part of a stem), a leaf disc and an inflorescence (with 4–5 open and several closed flowers) of similar diameter, or an inflorescence and two green fruits (siliques, ca. 14 mm long and 2 mm in diameter) (*n* = 14–20 per choice and stage or sex, depending on availability). Tests always started at noon. After 4 h, the consumed amount of material (in mm^2^) was estimated using graph paper. Each larva was only tested once. For adults, some went into all three tests due to a lack of availability, but had at least one or two days on their regular diet between each test. They were offered test combinations randomly in a different order. In tests with females, the number of eggs laid on flowers or fruits was also counted to test whether these plant parts are accepted at all for oviposition.

### Performance Experiment

To test the impacts of a single diet versus diet switching on the development, neonate larvae were separated into four treatment groups and reared in individual Petri dishes lined with moistened filter paper. Two groups were exclusively reared on either leaves (mix of leaves of different age, L), or flowers (mix of flower buds and open flowers, F), the other two groups received first only leaves and then from day five (L5F) or day 10 (L10F) after hatching only flowers (*n* = 40 per group). Fruits were not included as they were hardly accepted in the choice experiments. Larvae were weighed on the day of larval hatching, day 5 and day 10 after hatch and adults were weighed on the day of adult eclosion (microbalance ME36S, Sartorius AG Göttingen, Germany; 0.01 mg). The food was refreshed every other day and supplied *ad libitum*. Survival, day of pupation and day of adult eclosion were monitored daily. The sex of adult individuals was determined after the cuticle had hardened. Eight days after adult hatching, one female and one male of the same group were placed together in a Petri dish for 48 h, allowing for mating. Afterwards, females were placed back in individual dishes, and the number of eggs laid within the subsequent 96 h on the plant parts was counted. The experiment ended 48 days after larval hatching.

### Glucosinolate Analysis

To test for potential differences in the glucosinolate concentration and composition between the different plant organs, two young leaflets, two old leaflets, five flowers and three fruits were sampled from different plant individuals (*n* = 7–8 replicates) and shock-frozen in liquid nitrogen. After lyophilization, samples were weighed, homogenized, and extracted three times in 80% methanol, adding sinigrin at the first extraction as internal standard, as described in Barber and Müller (2021). After centrifugation, supernatants were applied on anion exchange columns [Sephadex A25 (AppliChem, Darmstadt, Germany), in 0.5 M acetic acid buffer, pH = 5.0], and columns were washed with water followed by 0.02 M acetic acid buffer (pH = 5.0). For conversion of glucosinolates to desulfoglucosinolates, *Helix pomatia* sulfatase (Sigma-Aldrich; in 0.02 M acetic acid buffer, purified as in Graser et al. 2001) was applied to the columns. One day later, desulfoglucosinolates were eluted with Millipore water, dried and resolved in water. The desulfoglucosinolates were analyzed via HPLC (Dionex Ultimate 3000, Thermo Fisher Scientific, Waltham, MA, USA) coupled with a diode-array detector (210–370 nm) with a Supelcosil LC18 reversed phase (150 × 3 mm, 3 μm) column (Supelco, Bellefonte, PA, USA), at 25 °C column temperature. A gradient of eluent A (Millipore water) and eluent B (methanol) at a flow rate of 0.35 mL min^− 1^ was used, starting at 5% B, held for 6.0 min, increasing to 38% B until 12.2 min, to 50% B until 14.3 min, to 60% until 16.6 min and to 90% B until 21.0 min, followed by a column cleaning and equilibration cycle. Glucosinolates were identified by comparing their retention times and spectra to an in-house database. For quantification, peak areas were quantified at 229 nm, related to those of the internal standard, to the response factors of 0.94 for 2-phenylethyl glucosinolate, 1 for other aliphatic glucosinolates, and 0.5 and 0.26 for aromatic and indolic glucosinolates, respectively, and further to the individual sample dry mass.

### Statistical Analysis

Statistical analyses were performed with R version 4.4.2 (R Core Team 2024). The data from the preference experiments were analyzed with the Wilcoxon test for paired samples as they were not normally distributed and the feeding on both offered plant parts was a paired response. Wilcoxon effect sizes (wef) were calculated by dividing the Z-statistic by the square root of the sample size with 95% confidence intervals being based on percentiles and estimated using 1000 iterations [package: rstatix (Kassambara 2023)]. The body mass at hatching, and at d 5 and d 10 after larval hatch, as well as the developmental time were analyzed for both sexes together using non-parametric Kruskal-Wallis tests with post-hoc Dunn-tests using Holm *p*-value adjustment to correct for multiple testing. Despite some of the data were normally distributed, this approach was taken to keep the test procedure consistent across developmental traits. For the Kruskal-Wallis effect sizes (kef) the eta-squared measure was calculated with bootstrap settings identical to the wef. The same statistical testing procedure was applied to data for the body mass of adult males and females separately as well as the total number of eggs laid within 96 h.

A survival analysis [packages: survival (Therneau 2024), survminer (Kassambra et al. 2024)] was performed to compare the survival (until day 48) of the individuals between the four treatment groups. Kaplan-Meier survival curves were plotted and differences between the curves tested with a log-rank test. The relative risk was calculated based on the survivability at day 48, with the confidence intervals being calculated using Wald’s normal approximation [package: DescTools (Signorell 2025)].

Total glucosinolate concentrations of the four plant parts were compared with a Kruskal-Wallis test followed by a post-hoc Dunn-test using Holm *p*-value adjustment to correct for multiple testing. Kruskal-Wallis effect sizes (kef) were calculated in the same way as for the insect developmental traits. The glucosinolate composition was analyzed after Wisconsin double standardization of square root-transformed data, using Kulczynski distance matrices [package: vegan (Oksanen et al. 2022)] and plotted with non-metric multidimensional scaling (NMDS). Comparison between the glucosinolate composition among the organs was performed using permutational multivariate analyses of variance (PERMANOVAs) and calculations of the multivariate dispersion, with 10,000 permutations.

## Results

### Preference Behavior of Larvae and Adults

**Fig. 1:**
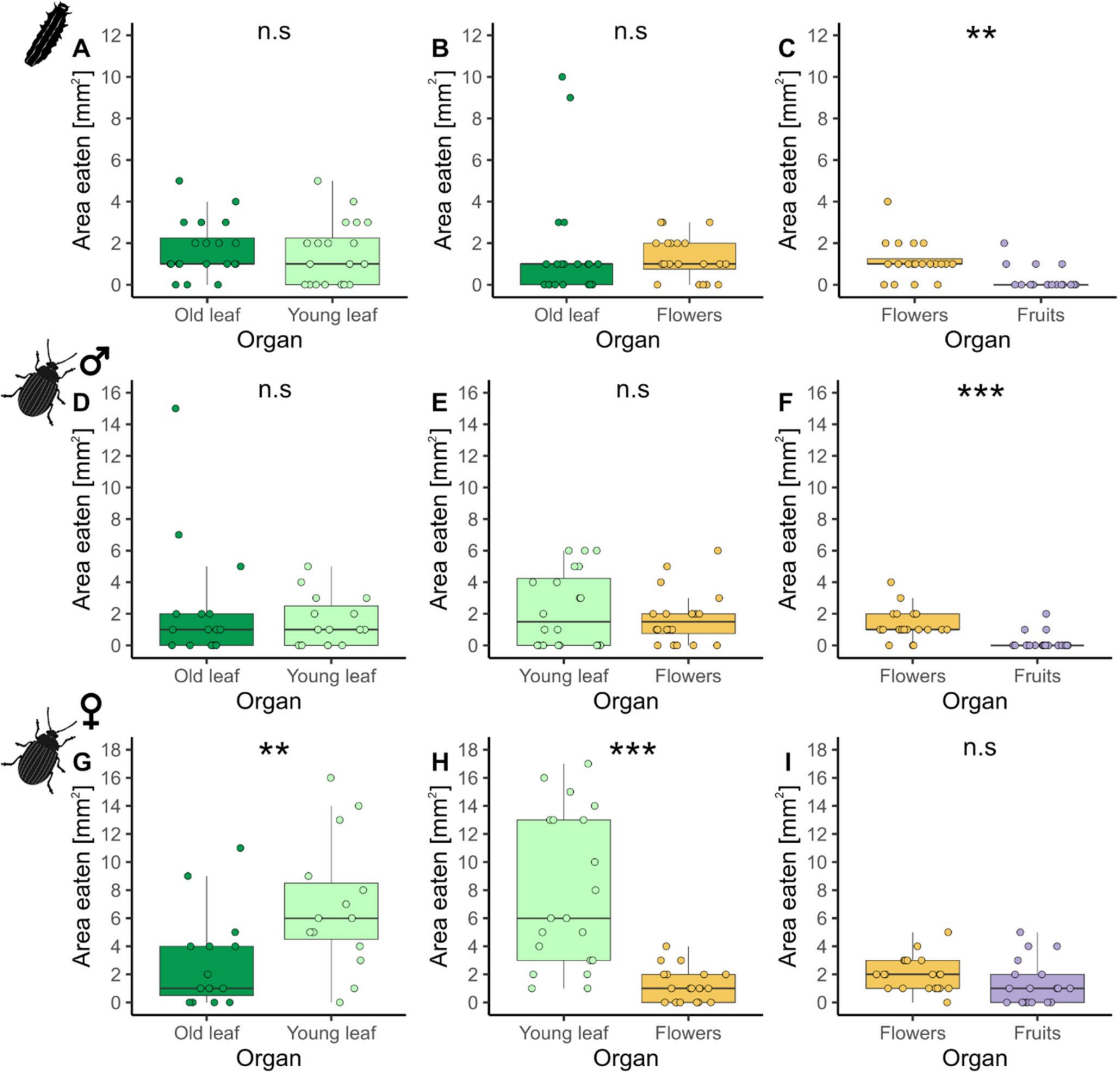
Feeding amounts of larvae (8 d after hatching; A-C) and adult males (D-F) and females (G-I) (39 d old) of *Phaedon cochleariae* in dual choice assays on different plant parts of*Nasturtium officinale* (**A**, **D**, **G**: old vs. young leaf, **B**, **E**, **H**: leaf vs. flower and **C**, **F**, **I**: flower vs. fruit). Results are presented as box-whisker plots showing medians (line), interquartile ranges (IQR, boxes) and the whiskers (extending to a maximum 1.5 x IQR) and individual data points plotted as dots. Wilcoxon test n.s. – not significant, ** *P* ≤ 0.01 and *** *P* ≤ 0.001

Larvae did not discriminate in their feeding between young and old leaves (wef = 0.089 [0.009, 0.500] 95% CI) or leaves and flowers (wef = 0.103 95% CI [0.005, 0.540]), but significantly preferred to feed on flowers over fruits (wef = 0.681 [0.400, 0.870] 95% CI), (Fig. 1A-B). Only 20% of the larvae (4 out of 20) fed on fruits to a small extent (Fig. 1C). Adult males showed a similar preference behavior as the larvae (wef young vs. old leaves = 0.022 [0.007,0.560] 95% CI; wef leaves vs. flowers = 0.165 [0.009, 0.560] 95% CI; wef flowers vs. fruits = 0.835 [0.730, 0.900] 95% CI), with only 3 out of 20 feeding on fruits (Fig. 1D-F). In contrast to larvae and adult males, adult females significantly preferred feeding on young versus old leaves (wef = 0.768 [0.510, 0.880] 95% CI), and on leaves over flowers (wef = 0.819 [0.660, 0.880] 95% CI), but they did not prefer flowers over fruits (wef = 0.408 [0.030, 0.750] 95% CI) (Fig. 1G-I). Few eggs were also found on flower petioles, but only very rarely on fruits.

### Performance in Dependence of Diet

**Fig. 2:**
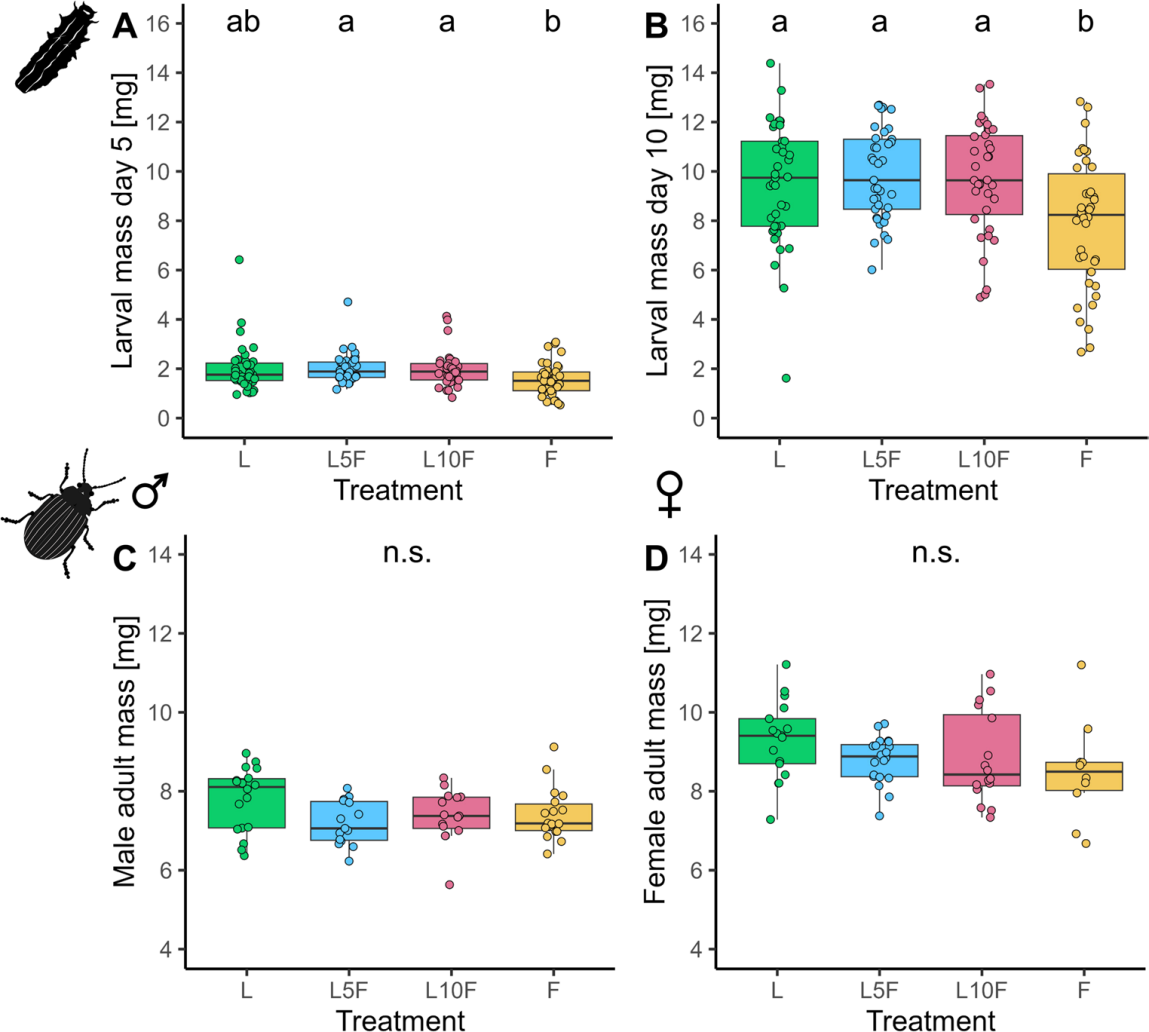
Body mass of larvae at day 5 (**A**), day 10 (**B**), and of adult males (**C**) and females (**D**) of *Phaedon cochleariae*, reared either exclusively on leaves (L), for the first 5 d or 10 d on leaves and then on flowers (L5F, L10F), or on flowers only (F). Results are presented as box-whisker plots showing medians (horizontal lines), means (red crosses), interquartile ranges (IQR with 25th and 75th percentiles; boxes), whiskers extending to data points within 1.5 x the IQR and raw data points. Different lowercase letters indicate significant differences between data, tested with Kruskal-Wallis tests followed by Dunn-tests with Holm p-value adjustment

Before distributing the larvae to the treatments, the initial larval body mass after hatching did not differ significantly (*Chi*^*2*^ = 0.19, kef = −0.018 [−0.020, 0.040] 95% CI, *df* = 3, *p* = 0.979, *n* = 40 per treatment), highlighting identical starting conditions. After 5 d of feeding in the different treatment groups, most larvae kept on leaves (groups L5F and L10F) were significantly heavier than individuals kept exclusively on flowers (group F) (*Chi*^*2*^ = 13.13, kef = −0.068 [0.005, 0.200] 95% CI, *df* = 3, *p* = 0.979, *n* = 40 per treatment) (Fig. 2A). After 10 d, all larvae that fed on leaves (L, L5F and L10F) at least for some time during the assay had a significantly higher larval mass than those that fed on flowers (*Chi*^*2*^ = 12.98, kef = 0.069 [0.009,0.210] 95% CI, *df* = 3, *p* = 0.005, *n* = 35–39 per treatment) (Fig. 2B). At adult emergence, males (*Chi*^*2*^ = 6.21, kef = 0.052, [−0.030, 0.290] 95% CI, *df* = 3, *p* = 0.102, *n* = 15–18 per treatment) and females (*Chi*^*2*^ = 5.31, kef = 0.039 [−0.030, 0.270] 95% CI, *df* = 3, *p* = 0.15, *n* = 10–20 per treatment) showed no significant differences in their body mass among treatment groups (Fig. 2C, D).

**Fig. 3:**
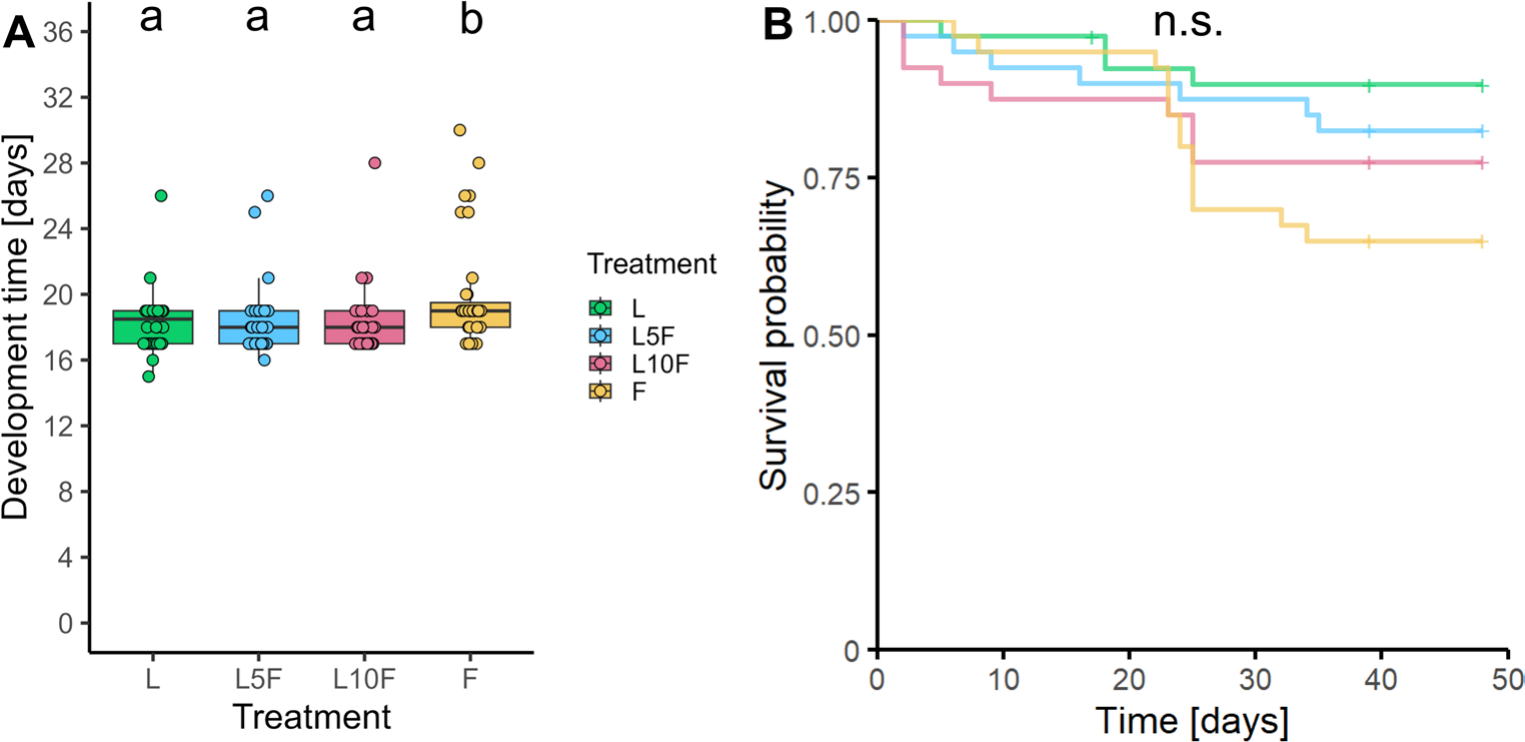
Developmental time from larval hatch until adult emergence (**A**) and survival probability of *Phaedon cochleariae, *reared either exclusively on leaves (L), for the first 5 d or 10 d on leaves and then on flowers (L5F, L10F), or on flowers only (F). Results of **A**) are presented as box-whisker plots showing medians (horizontal lines), means (red crosses), interquartile ranges (IQR with 25th and 75th percentiles; boxes), whiskers extending to data points within 1.5 x the IQR and raw data points. Results of **B**) are shown as Kaplan-Meier curves, starting at larval hatching (n = 40 per treatment group) and lasting until day 48 of the experiment, when it was ended. Short vertical lines represent right-censoring of individuals excluded from the analysis. Different lowercase letters indicate significant differences between data, tested with Kruskal-Wallis tests followed by Dunn-tests with Holm p-value adjustment (**A**) and log rank tests (n.s. not significant) (**B**)

The developmental time from larval hatch to adult emergence (both sexes pooled) differed significantly among all groups (*Chi*^*2*^ = 14.64, kef = 0.084 [0.020, 0.220] 95% CI, *df* = 3, *p* = 0.002, *n* = 35–36), being prolonged by 1–2 d in individuals of treatment group F (flowers only) (Fig. 3A). The survival probability did not differ among all groups (*df* = 3, *p* = 0.07), but individuals of treatment group F had by trend the lowest survival probability (Fig. 3B). In relation to the treatment group L, the relative risks of the L5F, L10F and F groups were 1.75 [0.59, 5.17] 95% CI, 2.25 [0.80, 6.32] 95% CI and 3.50 [1.34, 9.16] 95% CI, respectively.

**Fig. 4:**
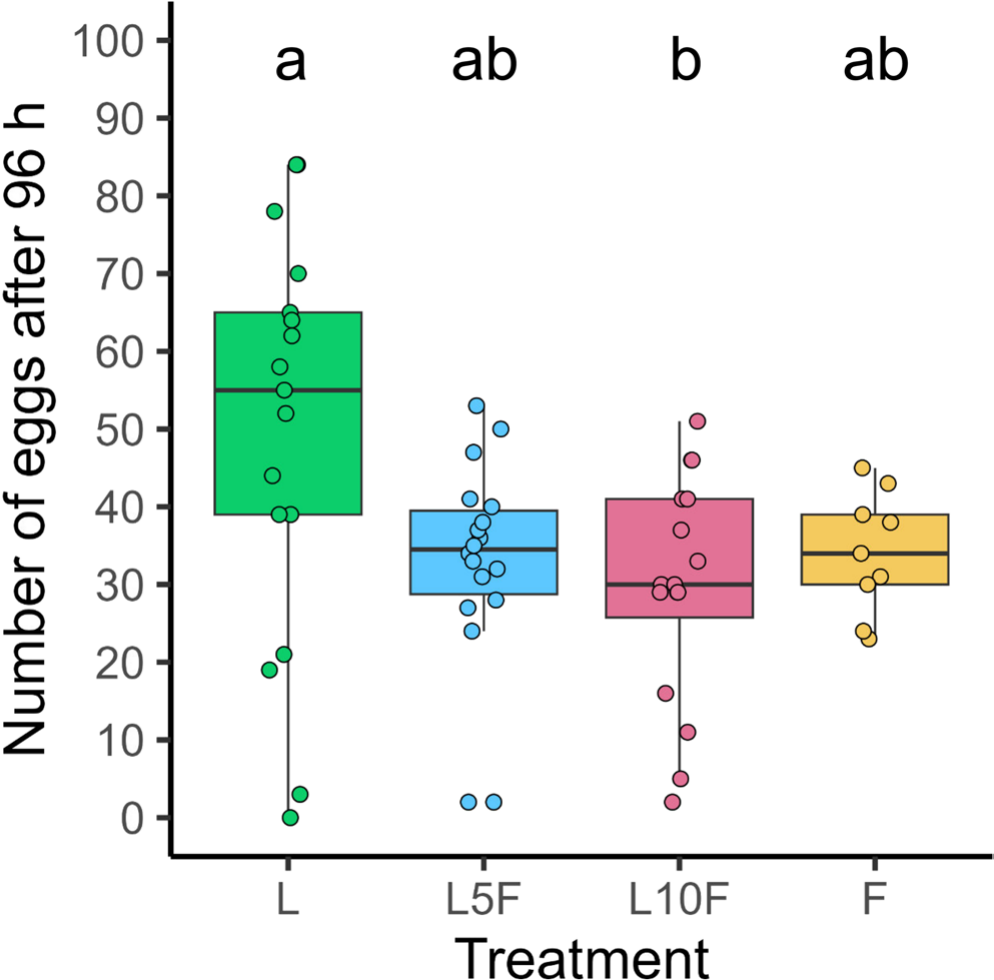
Short-term fertility of adult *Phaedon cochleariae*, reared either exclusively on leaves (L), for the first 5 d or 10 d on leaves and then on flowers (L5F, L10F), or on flowers only (F). Egg numbers were taken from day 10 after adult emergence for the subsequent 96 h. Results are presented as box-whisker plots showing medians (horizontal lines), means (red crosses), interquartile ranges (IQR with 25th and 75th percentiles; boxes), whiskers extending to data points within 1.5 x the IQR and raw data points. Different lowercase letters indicate significant differences between data, tested with a Kruskal-Wallis test followed by a Dunn-test with Holm *p*-value adjustment

Short-term fertility differed significantly among the females of the four treatment groups (*Chi*^*2*^ = 8.60, kef = 0.100 [−0.030, 0.400] 95% CI, *df* = 3, *p* = 0.035, *n* = 9–17). Females having been reared on leaves exclusively laid approx. 59–83% (median) more eggs than females of the other three groups, with a significant difference only to the L10F group (Fig. 4).

### Glucosinolate Composition in Different Plant Parts

**Fig. 5:**
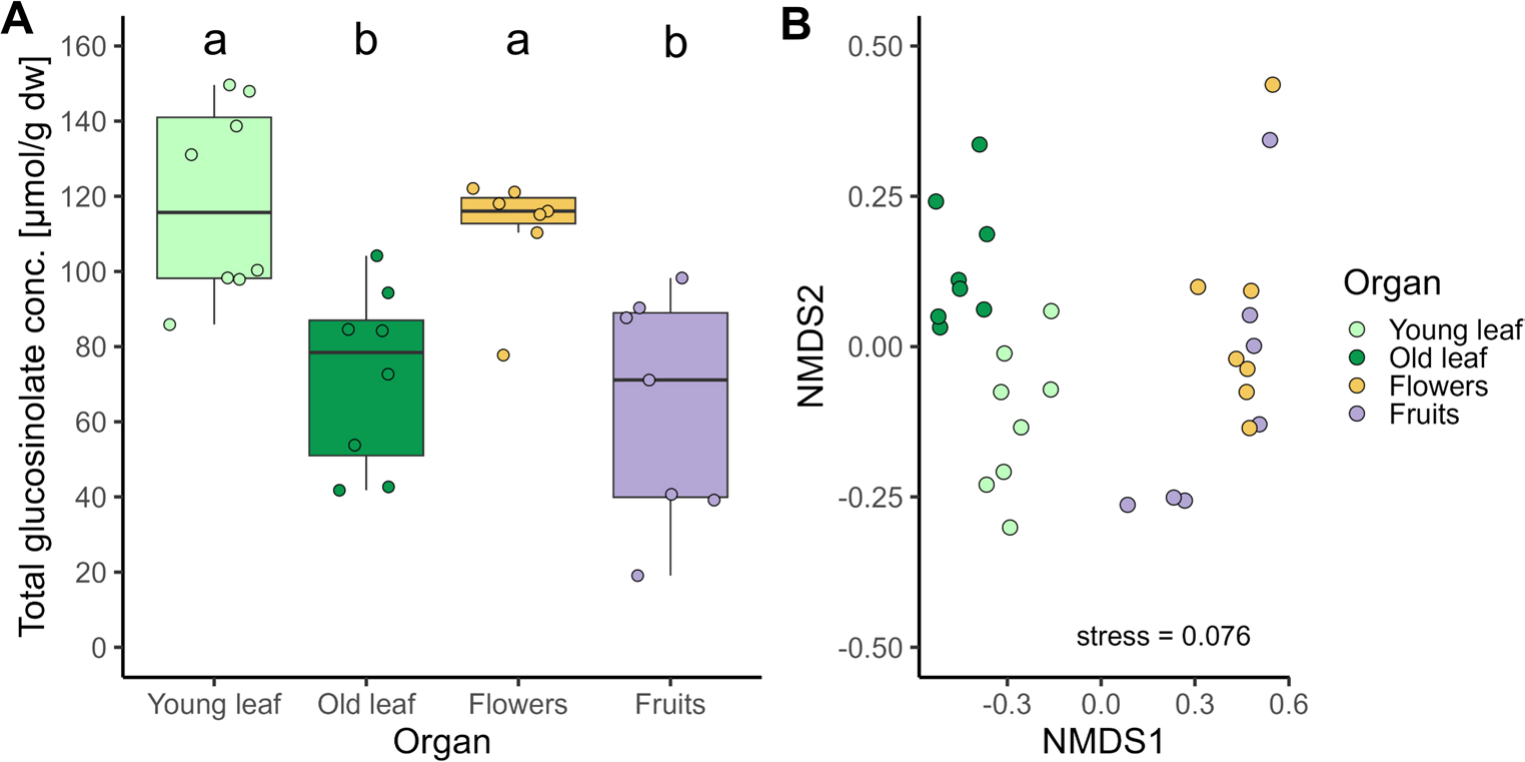
Total glucosinolate concentration (conc.) (**A**) and composition of ten glucosinolates (**B**) in young and old leaves, flowers and fruits of *Nasturtium officinale*. Results of (**A**) are presented as box-whisker plots showing medians (horizontal lines), means (red crosses), interquartile ranges (IQR with 25th and 75th percentiles; boxes), whiskers extending to data points within 1.5 x the IQR and raw data points. Different lowercase letters indicate significant differences between data, tested with a Kruskal-Wallis test followed by a Dunn-test with Holm *p*-value adjustment. Results of (B) are presented as NMDS, plotted using Kulczynski-distance after data were transformed using Wisconsin double standardization of square root transformed data; *n* = 7–8 per organ

The total glucosinolate concentration was significantly higher in young leaves and flowers than in old leaves and fruits of *N. officinale* (*Chi*^2^ = 16.21, *kef* = 0.508 [0.260, 0.750] 95% CI, *df* = 3, *p* = 0.001, *n* = 7–8) (Fig. 5A). Ten glucosinolates were found, with 2-phenylethyl glucosinolate being dominant in all organs, followed by 8-(methylsulfinyl)octyl glucosinolate, while 4-hydroxy-indol-3-ylmethyl glucosinolate was least abundant, not measurable at all (or below the detection limit) in old leaves. The overall composition differed significantly among treatment groups (ADONIS *R*^*2*^ = 0.75, *df* = 3, *p* < 0.001). Young and old leaves had distinct compositions and were strongly separated from the composition of flowers and fruits. The reproductive parts, flowers and fruits, showed partly overlapping glucosinolate compositions (Fig. 5B).

## Discussion

Usually considered as leaf herbivores, larvae and adults of *P. cochleariae* were found to accept not only leaves but also flowers for feeding, and adult females even accepted fruits. Moreover, *P. cochleariae* individuals could fully develop into adults when feeding exclusively on flowers, while their developmental performance was reduced on that diet.

Larvae and adult males of *P. cochleariae* did not differentiate between leaves of different age and leaves and flowers, but clearly avoided feeding on fruits in choice tests. In contrast, adult females showed a distinct preference behavior, preferring young over old leaves, young leaves over flowers, and also accepting fruits. This finding is in slight contrast to earlier studies, where both males and females were found to prefer young (green) over old (yellow) leaves of *N. officinale* (Kühnle and Müller 2011). In the previous study, leaves were offered for 20 h, not just 4 h as in the present study, and old leaves were more mature (yellow) than in the present test, which may explain the different outcome for the males. The young leaves may be preferred at least by the females, since they have a higher protein content and/or lower water content compared to old leaves of *N. officinale* (Reifenrath and Müller 2009). In addition, since glucosinolates are known to act as feeding stimulants for *P. cochleariae* (Nielsen 1978; Reifenrath and Müller 2008), higher glucosinolate concentrations in young compared to old leaves may turn the young leaves more attractive. In the field, *N. officinale* plants grow constantly and provide leaves of different age as well as flowers and fruits simultaneously from end of May until end of July (Düll and Kutzelnigg 2016). In *Phaedon brassicae*, individuals were observed in the field to feed on flowers and flower buds of *Arabidopsis halleri*, but not on developing fruits (Kawagoe and Kudoh 2010). The authors argue that fruits may be too tough (Kawagoe and Kudoh 2010), which could also apply to the fruits of *N. officinale* and *P. cochleariae*. In our choice assays, eggs of *P. cochleariae* were occasionally laid on flower petioles, but very rarely on fruits of *N. officinale*. Females lay their eggs in small cavities, which they bite into the tissue (Müller and Rosenberger 2006). Using other plant parts such as flowers and fruits for feeding and oviposition may provide an alternative nutritional source or oviposition substrate and, in the wild, also offer another niche to avoid competition or occupy an enemy-free space (Smallegange et al. 2007). An enemy-free space would be realized, if, for example, parasitoids use leaf synomones to find their prey but are not able to use compounds released from flowers attacked by the beetles. However, more research is needed in this direction, as to our knowledge little is known about parasitoids of *P. cochleariae*.

Changes in preferences for certain plant organs over the course of the insect development were found in different species, such as *Pieris brassicae* on *Brassica nigra* and *Athalia rosae* on *Sinapis alba*, where larvae move from the leaves to the flowers in the middle of the larval period (Bandeili and Müller 2010; Smallegange et al. 2007). These insect species even gained a higher body mass when feeding on flowers only. A higher pupal mass when reared on flowers compared to leaves of an Apiaceae was also found in *Depressaria leptotaeniae* (Lepidoptera: Oecophoridae), at least in laboratory trials (Thompson 1983). These patterns were different in *P. cochleariae*. Here, exclusive flower feeding prolonged the development and slightly, but not significantly, reduced survival. A potentially decreased feeding rate on this suboptimal host may have led to the decreased performance, as shown for larvae of *Pyrrhalta viburni* (Desurmont and Weston 2014). In contrast, larvae that fed on leaves during their development, irrelevant if exclusively or sequential, developed faster and reached a higher body mass as larvae. Nevertheless, more than 50% of the individuals forced to florivory reached adulthood and females laid comparable numbers of eggs as females that had been kept on a sequential diet of leaves and flowers. These findings indicate that florivory may be an alternative strategy for *P. cochleariae,* which individuals may use in nature especially when populations reach high densities and most of the leaves have been eaten. With regard to fertility, feeding on flowers may provide less nutrients required for oviposition to the females, reducing their capacity to lay eggs. Moreover, flowers wilted somewhat faster than leaves in the laboratory, which may have contributed to the overall lower numbers of eggs laid on flowers in the three treatment groups L5F, L10F, and F compared to females of the group that laid its eggs on leaves (L). However, on intact plants flowers may serve as suitable alternative substrate for oviposition, as females laid eggs on flowers. Movement of herbivores from leaves to flowers can be hindered by mechanical barriers, such as waxy surfaces of the petioles or trichomes (Abdalsamee and Müller 2015). On *N. officinale*, petioles do not show any such barriers and larvae can move up readily from the leaves to the inflorescences.

Individuals that experienced a switch in diet from leaves to flowers at different timepoints during the larval stage had a slightly faster development and a slightly (though not significant) higher survival, but showed a low short-term fertility, comparable to those individuals feeding exclusively on flowers. Thus, mixing of an optimal diet with a suboptimal diet can be to some extent more advantageous than feeding exclusively on a suboptimal diet. In contrast, in adult flies of *Liriomyza trifolii* lifetime fecundity could be enhanced significantly when they were kept on a mixed diet compared to exclusive feeding on either leaves or flowers (Merwin and Parrella 2014). Constant diet mixing, where nutrients of both organs can be used and potential toxins diluted as needed, may result in different outcomes than when different organs are offered in a sequence. Moreover, the impacts of florivory versus folivory on development are not only insect species-specific, but also host plant species-specific. While *A. rosae* developed faster when feeding on flowers than on leaves of *S. alba* (Bandeili and Müller 2010), they showed a slower development and gained less body mass when feeding on flowers of *Brassica nigra* (Abdalsamee and Müller 2015). Findings may also differ for *P. cochleariae* depending on the host plant species.

Glucosinolate concentrations were significantly higher in young leaves and flowers than in old leaves and fruits of *N. officinale*. Both young leaves and flowers may be equally important for the plant and, as highly valuable tissues, be optimally protected chemically, while fruits tend to be mechanically defended due to their toughness. In contrast, in other Brassicaceae, such as *Brassica nigra*, manifold higher glucosinolate concentrations were found in flowers compared to leaves (pool of all leaves) (Chrétien et al. 2022), while young leaves and flowers of *S. alba* had comparable concentrations, which were at least twice as high as in old leaves (Bandeili and Müller 2010). As *P. cochleariae* is well adapted to the glucosinolates and can conjugate their potentially toxic breakdown products with amino acids (Barber et al. 2024; Friedrichs et al. 2020), differences in concentration of glucosinolates among the different plant organs likely had no or little impact on the development of this insect species. These differences in glucosinolate concentration and also composition between young and old leaves and reproductive organs of *N.officinale*, as well as differences in myrosinase activities, which have previously been shown to be much higher in young compared to old leaves of this plant species (Reifenrath and Müller 2007) and may also differ between leaves and flowers, may still impact the preferences and performance of *P. cochleariae*. Apart from differences in the glucosinolate-myrosinase system, differences in various other (groups of) specialized metabolites, such as essential oils (Amiri 2012) or polyphenolics (Kyriakou et al. 2022), and in enzyme activities between the plant organs may impact the outcome of florivory versus florivory. Plant chemicals, such as the amounts of antioxidants, could also impact the immune response of insects, as shown in the arctiid *Parasemia plantaginis* (Ojala et al. 2005). Furthermore, different plant parts may be colonized by different microbes, which can impact the insects either indirectly by modulating the diet chemistry or directly by stimulatory acting metabolites of the microbes (Eberl et al. 2020). If insects were forced to feeding on flowers over several generations due to intense competition or other ecological reasons, individuals that cope well with this novel diet may be selected. Individuals may also differ in the extent to which they mix their diet (Bernays and Bright 1993).

In conclusion, the results from this study revealed that *P. cochleariae* develops best on an exclusive leaf diet. However, individuals are capable of incorporating other, less optimal plant organs into their diet, which can occur in nature when leaves are senescing, competition becomes high, or in response to pressure from symbionts such as gut-infesting gregarines (Wolz et al. 2022) or predators. Under such circumstances, the dietary flexibility may be beneficial, whereas in times of high availability of leaves, florivory may be disadvantageous due to the prolonged developmental time and reduced fertility. Thus, depending on the context, diet switching can be beneficial, but has its disadvantages. Further research is needed to determine conditions under which (supplementary) florivory is most beneficial and to explore this understudied topic also in other herbivorous species generally considered as folivores.

## Data Availability

A GitLab repository including a snapshot of used packages including their respective versions used can be found under https://gitlab.ub.uni-bielefeld.de/dozi/phaedon-dietswitch.
